# Disparities in children’s vocabulary and height in relation to household wealth and parental schooling: A longitudinal study in four low- and middle-income countries

**DOI:** 10.1016/j.ssmph.2017.08.008

**Published:** 2017-09-01

**Authors:** Sarah A. Reynolds, Chris Andersen, Jere Behrman, Abhijeet Singh, Aryeh D. Stein, Liza Benny, Benjamin T. Crookston, Santiago Cueto, Kirk Dearden, Andreas Georgiadis, Sonya Krutikova, Lia C.H. Fernald

**Affiliations:** a School of Public Health, University of California, Berkeley, CA, USA; b Department of Epidemiology, Harvard T.H. Chan School of Public Health, Boston, MA, USA; c School of Arts and Sciences, University of Pennsylvania, Philadelphia, PA, USA; d University College London, UK; e Rollins School of Public Health, Emory University, Atlanta, GA, USA; f Young Lives, Department of International Development, University of Oxford, UK; g College of Life Sciences, Brigham Young University, Provo, UT, USA; h Group for the Analysis of Development and Pontificia Universidad Católica del Perú, Lima, Peru; i IMA World Health, Dar es Salaam, Tanzania; j EDePo, Institute for Fiscal Studies, London, UK

## Abstract

Children from low socio-economic status (SES) households often demonstrate worse growth and developmental outcomes than wealthier children, in part because poor children face a broader range of risk factors. It is difficult to characterize the trajectories of SES disparities in low- and middle-income countries because longitudinal data are infrequently available. We analyze measures of children’s linear growth (height) at ages 1, 5, 8 and 12y and receptive language (Peabody Picture Vocabulary Test) at ages 5, 8 and 12y in Ethiopia, India, Peru and Vietnam in relation to household SES, measured by parental schooling or household assets. We calculate children’s percentile ranks within the distributions of height-for-age z-scores and of age- and language-standardized receptive vocabulary scores. We find that children in the top quartile of household SES are taller and have better language performance than children in the bottom quartile; differences in vocabulary scores between children with high and low SES are larger than differences in the height measure. For height, disparities in SES are present by age 1y and persist as children age. For vocabulary, SES disparities also emerge early in life, but patterns are not consistent across age; for example, SES disparities are constant over time in India, widen between 5 and 12y in Ethiopia, and narrow in this age range in Vietnam and Peru. Household characteristics (such as mother’s height, age, and ethnicity), and community fixed effects explain most of the disparities in height and around half of the disparities in vocabulary. We also find evidence that SES disparities in height and language development may not be fixed over time, suggesting opportunities for policy and programs to address these gaps early in life.

## Highlights

• Vocab disparities of top & bottom SES quartiles are larger than height disparities.
• Height disparities are present by age 1y and persist as children age.
• Vocab disparities emerge early in life, but patterns are not consistent across age.
• Household characteristics & community FE explain most of the height disparities.
• Household characteristics & community FE explain around half of the vocab disparities.

## Introduction

Optimal development in early childhood is associated with better health, cognitive and language development, and achievement, concurrently and later in life (Grantham-McGregor et al., 2007, Hoddinott et al., 2013a, Martorell et al., 2010, Victora et al., 2008). More than 250 million children under 5y are at risk for not meeting their developmental potential due to living in extreme poverty and/or because they have experienced linear growth retardation (stunting) (Black et al., 2016, Lu et al., 2016); direct measures of cognitive child development have confirmed the magnitude of the problem (McCoy et al., 2016). Children living in poverty receive fewer household- and community-level investments (e.g., nutrition, health, education, and responsive stimulation) than children who do not live in poverty (Engle et al., 2011, Walker et al., 2011b). For these reasons, among others, the United Nations has included reducing inequality within and among countries as a key Sustainable Development Goal (Nino, 2016).

Developmental disparities between children from lower and higher socio-economic status (SES) households persist into adulthood. In low- and middle-income countries, child height is associated with adult skills, marriage partner quality, and labor market outcomes (Hoddinott et al., 2013b, Hoddinott et al., 2013a). Better early cognitive skills, including those related to language, are associated with higher labor market earnings and lower levels of risky behavior later in life (Gertler et al., 2014, Heckman et al., 2006, Walker et al., 2011a). Thus, investing in children through a range of interventions can affect children’s cognitive and physical development, and can have long-term implications in many other domains (Britto et al., 2016, Hoddinott et al., 2008).

While the persistence of health and cognitive disparities has been well-documented in high-income countries (Bradley and Corwyn, 2002, Case et al., 2002, Pavalko and Caputo, 2013), less longitudinal research has been done in low- or middle-income countries. Most literature to date has relied on cross-sectional data, requiring strong assumptions to be made in drawing inferences about changes with age. Moreover, existing studies often capture a limited age range (Fernald et al., 2012, Fernald et al., 2011, Ghuman et al., 2005, Paxson and Schady, 2007, Rubio-Codina et al., 2015a). Evidence from Colombia, the country with the widest age-range we found to have been studied cross-sectionally, supports the hypothesis that disparities in vocabulary initially widen with age but the supposed widening may halt in later childhood, as the disparities at 8.5y are of a similar magnitude to those at 4.5y (Bernal and Van Der Werf, 2011, Rubio-Codina et al., 2015b).

The limited available longitudinal research supports these cross-sectional findings. Data from Bangladesh (following children from age 0 until 5y) (Hamadani et al., 2014), Ecuador (following children from 3–5y until 10–12y) (Schady et al., 2015), Madagascar (following children 3–6y until 7–10y) (Galasso, Weber, & Fernald, 2017), Nicaragua (following children 3–6y until 6–9y) (Macours, Schady, & Vakis, 2012), and the Young Lives countries (Ethiopia, India, Peru, and Vietnam, following children from 5y until 8y) (Lopez Boo, 2016) provide some additional evidence that SES-related differences in cognitive child development scores increase throughout early childhood, flatten around 5–7y, and are constant through the remaining pre-pubertal years.

We are aware of only three studies that engage in cross-country comparisons. These studies apply the same method in differing contexts, an advantage over comparing multiple, individual-county studies. In one cross-sectional analysis looking at disparities in early child development in India, Indonesia, Peru and Senegal, within-country differences in length-for-age and child development scores by SES are evident as early as 3–23mo (Fernald et al., 2012). Findings from another study of children 3–6y in five Latin American countries (some with cross-sectional data and some with longitudinal data) align with the hypothesis that disparities in vocabulary widen at early ages, with little further change once children are in elementary school (Schady et al., 2015). A final longitudinal study examines children from the Young Lives Study Countries (Ethiopia, India, Peru and Vietnam) at 5 and 8y, and finds the magnitude of within-country SES differences in vocabulary to persist over time (Lopez Boo, 2016). Each paper finds consistent patterns but the sizes of the SES disparities vary by country.

The existing literature implies that vocabulary disparities attributable to SES widen in early childhood and persist into middle childhood, though this hypothesis has not been directly tested. Furthermore, most of the extant studies do not address disparities in height after early childhood. Stunting had been thought to be determined by 2–3y (Victora et al., 2008); however, recent research from the Young Lives Study indicates that HAZ may be influenced by SES as late as 8y (Lundeen et al., 2014, Schott et al., 2013) and is associated with improved cognitive outcomes in children who experience growth recovery compared to children who are persistently stunted (Crookston et al., 2013). Data from rural Gambia further illustrates increases in HAZ in adolescence (Prentice et al., 2013). Variation in adult height is strongly predicted by growth in the first years of life (Stein et al., 2010), and there is limited evidence of potential for nutritional interventions to impact on linear growth when delivered after age 2y (Roberts & Stein, 2017). Thus identifying common underlying determinants of disparities in growth and cognitive achievement is of value because, while sharing some important inputs like nutrition, they are not perfectly correlated and height and vocabulary are both associated with schooling, economic productivity, health and other outcomes later in the life cycle (Britto et al., 2016).

Our study extends the existing research, in particular building on the cross-country Young Lives Study (Lopez Boo, 2016). Our objective is to contribute to the literature with a longitudinal description of childhood disparities in height and vocabulary (a measure of cognitive achievement) associated with two measures of SES (household wealth and parental schooling). Height data are available at 1, 5, 8 and 12y and vocabulary data are available at 5, 8, and 12y. We hypothesize that SES-related disparities in height will widen between 1 and 5y and that SES-related disparities in both height and vocabulary will remain constant from 5 to 12y. Since many studies confirm that household and community covariates account for much of these SES gaps (Fernald et al., 2012, Fernald et al., 2011, Hamadani et al., 2014, Lopez Boo, 2014, Rubio-Codina et al., 2015a), in a non-causal analysis we describe the extent to which these covariates at age 1y account for the size of the SES-related disparities at 12y.

## Methods

### Data

We analyze data from the Young Lives Study, which recruited children in each of four countries (Ethiopia, India, Peru, and Vietnam) in 2002 (Barnett et al., 2013). The present analysis uses data from the younger cohort, who were between 6.0 and 17.9 months at recruitment (mean 11.7 months). Follow-up data were collected in 2006 (mean 5.3y), in 2009 (mean 7.9y), and in 2013 (mean 12.0y). We refer to the survey rounds as ages 1, 5, 8 and 12, respectively.

In each of the study countries, participants were selected through a multi-stage sampling process beginning with 20 sentinel sites that were purposively selected to reflect the Young Lives study’s aims of examining the causes and consequences of childhood poverty and diversity of childhood experiences. In India, recruitment was restricted to the state of Andhra Pradesh, which subsequently divided into two states, Andhra Pradesh and Telangana. Within each sentinel site, approximately 100 children within the eligible age category were randomly sampled (“Young Lives methods guide,” 2017). Less than 2% of selected households refused to participate. There was one study child per household. Comparisons with children in the nationally-representative Demographic and Health Surveys (DHS) found the Young Lives samples to cover a broad diversity of children within each country (“Young Lives methods guide,” 2017).

### Sample size and exclusions

The first Young Lives survey round (age 1y) included 1999 children in Ethiopia, 2011 children in India, 2052 children in Peru, and 2000 children in Vietnam. We limit the analytic sample to children for whom the following data are available: wealth index, parents’ or caregiver’s schooling, HAZ, and vocabulary test (and whether it was taken in the same major language at both 5 and 12y) (Table A1). Being a “major language” was defined as having at least 100 children take the test in that language in the 5 and 12y surveys. Because our analysis focuses on final outcomes at 12y, we do not restrict the sample used in the main analysis based on language of the test taken or availability of outcome data at 8y. Major languages by this definition are Amarigna (Amharic), Oromifa, and Tigrigna for Ethiopia; Telugu for India; Spanish for Peru; and Tiếng Việt for Vietnam. We include all major languages instead of official languages due to the large number of children in Ethiopia who took the vocabulary assessment in a range of languages. A robustness check considers only children who took the vocabulary assessment in Amarigna, the official language in Ethiopia. We drop observations with implausible values beyond six standard deviations for HAZ [Ethiopia N=1; India N=4; Peru N=3; Vietnam N=3]. Children in the analytic sample generally had higher measures of SES than those who were excluded (Table A2).

### Variables used to characterize SES

The household wealth index variable, measured at 1y, is country-specific. Details regarding variables included for each country and their weights are available elsewhere (Alemu et al., 2003, Escobal et al., 2003, Galab et al., 2003, Tuan et al., 2003). The wealth index includes measures of housing quality, ownership of consumer durables, and access to services such as electricity, water and sanitation; these sub-indices are weighted equally in the composite index. We divide the analytical sample within each country into quartiles based on the wealth index. Although Peru and Vietnam are higher income countries than Ethiopia and India, not all components of the wealth index reflect this difference. For example, all countries have close to universal coverage of electricity in the top quartile, but India’s lower quartile has higher electricity coverage than Peru’s (Table A3).

**Fig. A1:**
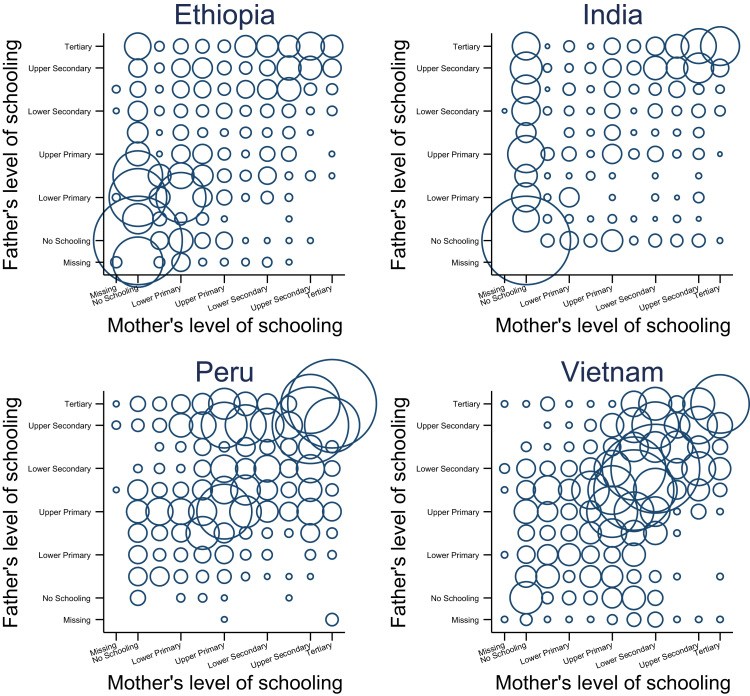
Mothers’ and Fathers’ Paired Schooling Levels. Labeled values are completed levels; intermediate levels are incomplete. The size of the circles illustrates the number of children with parents with those schooling levels.

Parental schooling was recorded when the child was 5y. We code parental formal schooling attainment according to country-specific thresholds of lower and upper primary and lower and upper secondary. Respondents who indicated that they were literate but had not participated in any formal schooling [Ethiopia N=219; India N=75; Peru N=1; Vietnam N=0] are assigned to the incomplete lower primary schooling level. Fig. A1 illustrates the distribution of parental schooling pairs and shows the schooling levels that are coded with integer values 0-9. Children with information on only one parent’s schooling [Ethiopia N=113; India N=1; Peru N=9; Vietnam N=25] are assigned the schooling level of that parent. Children with no information on parental schooling [Ethiopia N=4; India N=0; Peru N=0; Vietnam N=1] are assigned the schooling level of the caregiver. One child in Ethiopia did not have information on parental or caregiver schooling, so questions from the previous survey round (1y) regarding whether the caregiver and caregiver’s partner had completed primary or secondary school are used to assign parental levels of schooling. Using this average parental schooling index, we divide the analytical sample within each country, approximating quartiles as closely as possible.

Wealth refers directly to assets that are available, but parental schooling may also represent parental knowledge of good child development practices and the opportunity costs of time. In this study the correlation coefficients for household wealth and parental schooling are 0.64 for Ethiopia, 0.58 for India, 0.59 for Peru, and 0.65 for Vietnam.

### Child outcomes: height and language development

Supine length (at 1y) and height (at ages 5, 8, and 12y) were measured to 1 mm using standardized length boards and stadiometers. Height-for-age Z scores (HAZ) were computed using the WHO Growth Standards (World Health Organization, 2006) for children <60mo and the WHO Growth References (de Onis et al., 2007) for children >60mo. Length-for-age was measured at 1y, but for consistency with later height measurements, we refer to the length-for-age z-score as HAZ. Because HAZ of infants is inversely correlated with age in many low- and middle-income countries (Victora, de Onis, Hallal, Blössner, & Shrimpton, 2010), the 1y HAZ measurements are adjusted to their predicted value at age 12mo by calculating the difference between each child’s HAZ and the mean HAZ for children within 1 month of the child’s age in the same country. This value is added to the mean HAZ for children aged 11–13mo. This adjustment is preferable to adding age as a covariate in the model because the adjustment does not assume a linear relationship between HAZ and age. This technique has been employed in previous analyses (Andersen et al., 2015, Crookston et al., 2013, Lundeen et al., 2014).

The Young Lives Study data set includes several measures of cognition including vocabulary, reading, writing, and math, but vocabulary is the only test used here because it was consistently administered as early as age 5 years. The vocabulary test has a sufficient range in difficulty to be applied at all ages, which allows for increased confidence in the longitudinal comparisons of child cognition. Children were administered the Peabody Picture Vocabulary Test (PPVT) version 3 (Dunn & Dunn, 1997) and, in Peru, the Spanish Version (Test de Vocabulario en Imágenes Peabody) (Dunn, Padilla, Lugo, & Dunn, 1986) at 5, 8, and 12y. Country- and round-specific details about the test, including selection of questions, implementation, and psychometric properties, can be found elsewhere (Cueto and Leon, 2012, Cueto et al., 2009). To compare results over time, we age-normalized the raw scores within each survey round and language of administration. The means and standard deviations used to calculate the age and language standardized PPVT scores are generated applying a previously-used methodology: mean PPVT for the age in months is estimated with a cubic polynomial (Rubio-Codina et al., 2015a). For the age-conditional standard deviation, we square the residuals of the previous regression, and regress them on another cubic polynomial of age in months. This method allows for continuity in the standardized scores across months but still allows for flexibility by month of the mean and variance used in the standardization.

There is evidence that measures of child health and development vary in terms of how they are related to SES variables; for example, SES disparities in Madagascar are larger for vocabulary scores than for linear growth (Fernald et al., 2011). These comparisons are challenging because the growth and language processes are not often measured on the same scale. Thus we use percentiles, an approach used before in studies on skill comparison (Neal, 2006) and intergenerational mobility (Chetty et al., 2014, Zhang et al., 2014). In order to compare the two outcomes, we compute the percentile rank of each child on HAZ and age- and language-standardized PPVT. We also provide analyses using the raw HAZ distribution in the Appendix A. Because there is no global standard for vocabulary, we do not include standardized cross-country comparisons for language.

We use the interchangeable terms ‘disparity’ and `gap’ to refer to differences in mean percentile rank of height or PPVT score between top and bottom quartiles of the household wealth or parental schooling indices. Larger gaps arise from stronger associations between SES and child outcomes, which could be interpreted as inequality.

Correlations between the standardized height and vocabulary outcomes range from 0.11 to 0.26; correlations between the percentile ranks of the two outcomes are slightly higher, ranging from 0.17 to 0.37.

### Household covariates and community fixed effects

All covariates were recorded when children were 1y. Covariates include mother’s height in centimeters, mother’s age in years, ethnicity indicator variables,^1^ and an indicator variable for whether the mother speaks the region’s official language. We impute missing covariates (Table A4) using multivariate normal regression (20 repetitions; Stata command *mi impute mvn*). We use sentinel site location codes to generate community fixed effects. By 12y, 12% (Ethiopia), 14% (India), 75% (Peru) and 9% (Vietnam) of children no longer lived within the sentinel sites, thus we use the child’s community from age 1y to define these fixed effects.

### Statistical analyses

For each country (Ethiopia, India, Peru, Vietnam) measure of SES (wealth or parental schooling), and outcome (percentile height and percentile vocabulary), we graph the mean and 95% confidence intervals of each outcome at each age by SES quartile. We impute outcome variables missing at age 8y (Table A4) using multivariate normal regression (20 repetitions; Stata command *mi impute mvn*).

For each combination of country, SES measure, and outcome, we test for the presence of non-parallel linear trends in child age using the OLS regression

(1) $$y_{\mathrm{it}}=\beta_{Q}Q_{i1}+\sum_{\forall t\in j}\beta_{a}a_{t}+\beta_{p}Q_{i1}∙A_{\mathrm{it}}+C+e_{\mathrm{it}}$$

in which the outcome variable for each child i at age *t* is $y_{\mathrm{it}.}Q_{i1}$ is an indicator variable for child i being in the top SES quartile in early childhood (age 1 for wealth, age 5 for parental schooling) versus being in the bottom quartile. Children in middle quartiles are not included in this analysis. The coefficient on this variable, $\beta_{Q}$, measures the size of the disparity at the first age the outcome variable is measured, 1y for height and 5y for vocabulary. We control for time factors a that influence all children, measured by indicator variables for the age at each survey $a_{t}$. For HAZ, elements of *j* are 5, 8, and 12y; for vocabulary, elements of *j* are 8 and 12y. *C* is the mean outcome at the first age measured of the reference group, the children in the bottom quartile. To test for parallel trends, we examine $\beta_{p}$, the coefficient on the interaction between age and the variable that indicates the child was in the top SES quartile at age 1y. Age in the interaction term $A_{\mathrm{it}}$ is distinct from $a_{t}$, as $A_{\mathrm{it}}$ is continuous and $a_{t}$ are indicators. We reject the null hypothesis of parallel trends if $\beta_{p}$ is statistically distinct from 0. The error term is $e_{\mathrm{it}}$. We cluster standard errors at the child level. In a robustness check, we test whether disparities change over time in comparison to the disparity present at 1y (i.e., do the differences between high and low SES become more or less pronounced at each age). In no cases do we reject the assumption of monotonicity of the differences over age, so we present the simpler specification. To test that parallel trends do not arise from worsening scores for both top and bottom quartiles, we test that, for the lowest quartile, slopes are not negative.

To consider the sensitivity of the height findings at 12y to puberty progression, which is associated with SES (Deardorff et al., 2014, James-Todd et al., 2010), we calculate an expected increase in height within the top and bottom quartiles for those who, per self-report, did not yet have evidence of initiation of puberty (onset of menses in girls and voice-lowering in boys). We calculate mean percentile of HAZ by girls’ menstruation onset status and boys’ low voice status. Since research suggests that age of onset of puberty is not correlated or is weakly correlated with final height, we assume that children who did not yet exhibit these puberty markers would later achieve the mean height of those who did exhibit them (Limony et al., 2015, Lundeen et al., 2016, Stein et al., 2016, Vizmanos et al., 2001). We calculate the differences in mean height and multiply these differences by the portion of boys and girls respectively in each quartile who had not yet exhibited the puberty marker. We weight these final sex-specific adjustments by the portion of boys and girls in the analytic sample and report this final adjustment as a percentage of the disparity at 12y.

We examine the extent to which controlling for household variables and community fixed effects, separately and together, changes the magnitude of the disparities. We perform the following analysis twice: when the outcomes were first measured (1y or 5y) and at 12y. In both cases *Q*_*i*_ refers to top quartile in wealth or parental schooling as measured in early childhood.

(2) $$y_{i}=\beta_{Q}Q_{i}+C+v_{i}$$

The magnitude of the SES disparity at age t without any adjustment is given by $\beta_{Q}$ , *C* is the mean outcome of the reference group, the bottom quartile, and the error term is $v_{i}$. This coefficient is adjusted for the SES variable not being used to define the disparity (e.g. education is included as a covariate for the wealth models and wealth is included as a covariate for the education models). The coefficient is also adjusted for household-level covariates described above. We also adjust separately for initial community-level fixed effects, with communities defined as the sentinel sites in the Young Lives sampling framework. We choose to use age 1y location for the community fixed effects in spite of some subsequent moves because of the emphasis in the literature on the early years as being the most critical for height and cognition (Martorell et al., 2010, Victora et al., 2008). Finally, we examine the size of the gap after adjusting for both maternal characteristics and community fixed effects. For all regressions including household characteristics, we use multiple imputation estimates. All analyses were performed in Stata 14.

## Results

**Fig. 1:**
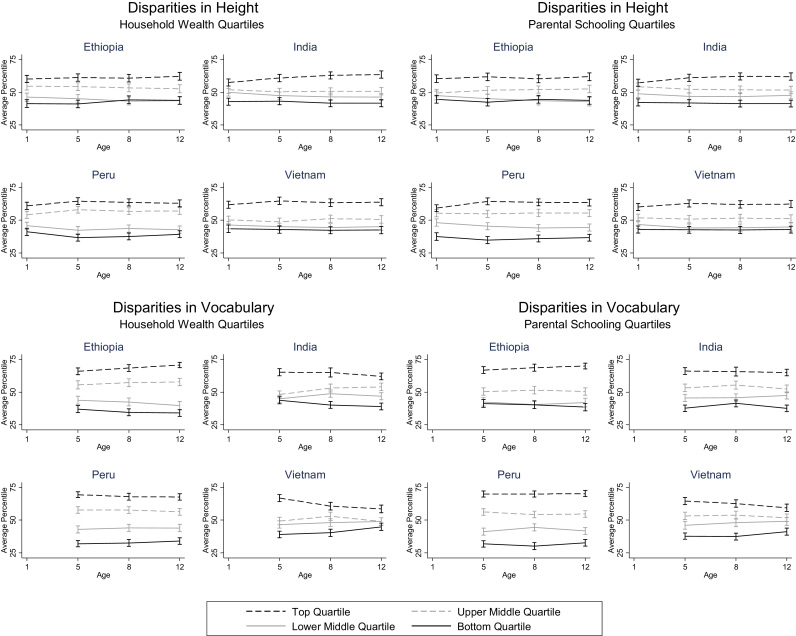
Disparities in height and vocabulary scores from household wealth and parental schooling, Young Lives study. Vocabulary (PPVT) is standardized within country and language of administration.

In all four samples at all ages, children living in households in the top quartile of SES are taller and have better language proficiency than those living in households in the bottom quartile of socio-economic status (Fig. 1). These results are consistent whether socio-economic status is defined by household wealth or by parental schooling (Table 1). On average, the difference in HAZ between a child from low and high SES households is about 1 SD; this translates into a 5cm height deficit for 5-year-old girls and a 7cm deficit for 12-year-old girls. The differences in average receptive vocabulary by top and bottom SES quartiles are approximately 1 standard deviation at both age 5 and age 12, with the exception of Vietnam, where the difference is around 0.4 standard deviations at age 12. These estimates cannot be converted to number of words known because there is not a cross-cultural, normative vocabulary scale, but they can give a general sense of effect size.

**Table 1:** 

**Panel A: Summary Statistics by Wealth Disparity**		**Ethiopia**				**India (AP & TG)**^b^				**Peru**				**Vietnam**			
		Bottom Quartile		Top Quartile		Bottom Quartile		Top Quartile		Bottom Quartile		Top Quartile		Bottom Quartile		Top Quartile	
	**Variables**	mean	sd	mean	sd	mean	sd	mean	sd	mean	sd	mean	sd	mean	sd	mean	sd
Disparities	Wealth Index	0.05	0.04	0.48***	0.09	0.16	0.06	0.68***	0.08	0.21	0.06	0.70***	0.05	0.19	0.08	0.72***	0.10
	Average Parental Schooling^a^	1.32	1.47	5.38***	2.69	1.77	2.22	6.34***	2.65	4.37	2.08	7.84***	1.40	3.59	1.87	6.81***	1.61
																	
Outcomes	Age & language standardized PPVT score age 5	-0.42	0.69	0.43***	0.97	-0.19	0.87	0.51***	1.23	-0.59	0.84	0.65***	0.88	-0.40	0.75	0.56***	1.11
	Age & language standardized PPVT score age 8	-0.53	0.70	0.61***	0.94	-0.30	0.78	0.55***	1.17	-0.50	0.98	0.64***	0.76	-0.28	0.79	0.43***	1.07
	Age & language standardized PPVT score age 12	-0.41	0.87	0.69***	0.50	-0.25	0.91	0.45***	0.75	-0.37	0.84	0.69***	0.76	-0.02	0.87	0.34***	0.69
	Height-for-age z-score age 1 (adjusted)	-2.29	1.78	-1.26***	1.61	-1.65	1.47	-1.01***	1.28	-1.53	1.21	-0.72***	1.21	-1.42	1.33	-0.71***	1.12
	Height-for-age z-score age 5	-1.71	1.05	-0.98***	0.98	-1.88	0.96	-1.29***	0.98	-1.91	0.98	-0.88***	0.97	-1.55	0.95	-0.75***	1.04
	Height-for-age z-score age 8	-1.31	0.97	-0.74***	0.97	-1.69	1.19	-1.04***	1.11	-1.46	0.98	-0.57***	0.96	-1.32	0.96	-0.54***	1.09
	Height-for-age z-score age 12	-1.65	0.92	-01***	1.00	-1.75	0.92	-0.97***	1.06	-1.32	1.00	-0.44***	0.98	-1.25	1.01	-0.43***	1.10
																	
Mother Variables	Mother Speaks Local Language Fluently	0.82	0.38	0.81	0.39	0.31	0.46	0.59***	0.49	0.11	0.32	0.08*	0.27	0.87	0.33	0.99***	0.09
	Mother's Age	28.33	6.78	26.89***	6.18	23.51	4.49	23.94	4.03	26.45	6.88	27.05	6.33	25.87	5.51	29.19***	5.78
	Mother's Height (cm)	157.69	5.95	159.49***	6.49	150.75	5.62	152.03***	5.21	149.06	5.70	151.99***	5.47	152.00	5.74	152.69*	5.49
																
	N	336		351		397		403		386		401		378		388	

**Panel B: Summary Statistics by Parental Schooling Disparity**		**Ethiopia**				**India (AP & TG)**^b^				**Peru**				**Vietnam**			
		Bottom Quartile		Top Quartile		Bottom Quartile		Top Quartile		Bottom Quartile		Top Quartile		Bottom Quartile		Top Quartile	
	**Variables**	mean	sd	mean	sd	mean	sd	mean	sd	mean	sd	mean	sd	mean	sd	mean	sd
Disparities	Wealth Index	0.16	0.11	0.42***	0.15	0.29	0.15	0.58***	0.18	0.29	0.14	0.59***	0.15	0.30	0.18	0.63***	0.18
	Average Parental Schooling^a^	0.00	0.00	6.8***	1.23	0.00	0.00	7.92***	0.82	2.91	1.05	8.77***	0.25	2.71	1.21	7.62***	0.89
																	
Standardized Outcomes	Age & language standardized PPVT score age 5	-0.30	0.81	0.49***	1.07	-0.37	0.64	0.56***	1.26	-0.60	0.81	0.67***	0.90	-0.42	0.76	0.51***	1.18
	Age & language standardized PPVT score age 8	-0.34	0.85	0.61***	0.94	-0.27	0.81	0.54***	1.13	-0.57	1.01	0.70***	0.74	-0.36	0.74	0.49***	1.06
	Age & language standardized PPVT score age 12	-0.29	0.95	0.66***	0.57	-0.31	0.94	0.53***	0.74	-0.43	0.86	0.76***	0.82	-0.12	0.90	0.36***	0.68
	Height-for-age z-score age 1 (adjusted)	-2.09	1.87	-1.26***	1.62	-1.71	1.60	-0.99***	1.47	-1.66	1.20	-0.8***	1.16	-1.44	1.19	-0.79***	1.23
	Height-for-age z-score age 5	-1.66	1.02	-0.94***	1.05	-1.94	0.88	-1.30***	1.03	-1.99	1.00	-0.89***	0.98	-1.56	0.90	-0.84***	1.03
	Height-for-age z-score age 8	-1.30	1.05	-0.76***	0.99	-1.75	0.95	-1.03***	1.10	-1.51	0.94	-0.58***	0.92	-1.31	1.00	-0.62***	1.06
	Height-for-age z-score age 12	-1.63	0.87	-1.02***	1.01	-1.76	0.95	-1.03***	1.06	-1.41	0.97	-0.44***	1.01	-1.25	1.03	-0.49***	1.11
																	
Mother Variables	Mother Speaks Local Language Fluently	0.88	0.33	0.82**	0.39	0.29	0.45	0.63***	0.48	0.09	0.29	0.09	0.29	0.88	0.33	0.99***	0.09
	Mother's Age	29.24	7.31	26.03***	5.48	24.32	5.03	23.41***	3.40	28.50	7.63	27.49**	5.82	26.17	6.11	29.35***	5.36
	Mother's Height (cm)	158.44	5.64	159.11	6.66	150.85	5.61	152.52***	5.31	148.36	5.66	152.55***	5.16	151.78	5.70	152.81***	5.57
																
	N	332		344		406		430		405		371		403		420	

Means of the Top Quartile and Bottom Quartile are significantly different at *10% **5% ***1%

Mother ethnicity indicators not listed.

a Levels are from 0 - 8: (0) no education (1) incomeplete lower primary (2) complete lower primary….(8) complete upper secondary.

b Andhra Pradesh and Telangana.

There are disparities in HAZ in all countries at 1y, and in the majority of cases, the size of the SES difference remains constant as children age (Table 2, Panel A). For Indian children, the height gap widens during childhood for both wealth and schooling, and for the Peruvian children the gap widens for schooling. At 5y, SES differences in vocabulary exist in all countries; in three cases for wealth and two for schooling, the disparity is not constant across age (Table 2, Panel B). The disparities in vocabulary widens with age in Ethiopia but narrows in Vietnam and Peru. The disparity in vocabulary from wealth also shrinks with age in Peru.

**Table 2:** SES disparities between top and bottom quartiles & testing for parallel trends.

	**Wealth Disparity**				**Schooling Disparity**			
	**Ethiopia**	**India**	**Peru**	**Vietnam**	**Ethiopia**	**India**	**Peru**	**Vietnam**
Panel A		**(AP & TG)**^a^				**(AP & TG)**^a^		
**Percentile Height: Ages 1, 5, 8, & 12**	Coef./SE	Coef./SE	Coef./SE	Coef./SE	Coef./SE	Coef./SE	Coef./SE	Coef./SE
Top Quartile Indicator Variable	19.106***	14.446***	22.567***	19.003***	16.394***	15.587***	23.963***	17.874***
(SES Gap at Age 1)	(2.01)	(1.92)	(1.92)	(2.05)	(2.04)	(1.91)	(1.91)	(1.98)
								
Age 5 Indicator Variable	0.631	0.462	-1.066	0.636	-0.617	0.574	0.581	0.819
	(1.24)	(1.02)	(0.98)	(0.78)	(1.16)	(0.98)	(0.98)	(0.80)
								
Age 8 Indicator Variable	2.229	-0.315	-1.479	-0.61	-0.441	0.113	0.209	0.185
	(1.39)	(1.20)	(1.03)	(1.05)	(1.28)	(1.13)	(1.06)	(1.02)
								
Age 12 Indicator Variable	2.875	-1.36	-1.504	-0.9	-0.306	-0.978	-0.382	0.054
	(1.76)	(1.46)	(1.32)	(1.31)	(1.56)	(1.34)	(1.32)	(1.27)
								
**Top Quartile (Indicator) X Age (Continuous)**	**-0.103**	0.693***	**0.274**	**0.252**	**0.12**	0.517***	0.385**	**0.183**
	**(0.22)**	**(0.18)**	**(0.17)**	**(0.16)**	**(0.21)**	**(0.17)**	**(0.16)**	**(0.16)**
								
Constant (Bottom Quartile at Age 1)	41.151***	42.552***	39.673***	43.047***	44.180***	41.699***	36.143***	42.487***
	(1.45)	(1.36)	(1.37)	(1.45)	(1.49)	(1.32)	(1.40)	(1.35)
								
p-value of coefficient on Top Quartile X Age	0.634	0	0.108	0.113	0.561	0.003	0.019	0.251
								
N	2744	3197	3138	3053	2699	3341	3092	3284

	**Wealth Disparity**				**Schooling Disparity**			
	**Ethiopia**	**India**	**Peru**	**Vietnam**	**Ethiopia**	**India**	**Peru**	**Vietnam**
		
Panel B		**(AP & TG)**^a^				**(AP & TG)**^a^		
**Percentile Vocabulary: Ages 5, 8, & 12**	Coef./SE	Coef./SE	Coef./SE	Coef./SE	Coef./SE	Coef./SE	Coef./SE	Coef./SE
Top Quartile Indicator Variable	24.175***	21.226***	40.028***	37.363***	21.287***	27.790***	39.242***	33.650***
(SES Gap at Age 5)	(2.90)	(3.12)	(2.39)	(3.12)	(3.12)	(2.99)	(2.53)	(3.01)
								
Age 8 Indicator Variable	-1.62	-2.397*	0.352	0.639	-0.872	2.094	-0.631	0.8
	(1.29)	(1.37)	(0.97)	(1.29)	(1.39)	(1.37)	(1.02)	(1.30)
								
Age 12 Indicator Variable	-2.896*	-4.626***	2.072*	5.826***	-2.818	-0.291	0.942	3.511**
	(1.62)	(1.65)	(1.23)	(1.61)	(1.85)	(1.68)	(1.38)	(1.60)
								
**Top Quartile (Indicator) X Age (Continuous)**	1.076***	**0.201**	-0.541**	-2.021***	0.850**	**-0.106**	**-0.101**	-1.250***
	**(0.30)**	**(0.33)**	**(0.24)**	**(0.34)**	**(0.33)**	**(0.33)**	**(0.26)**	**(0.33)**
								
Constant (Bottom Wealth Quartile at Age 5)	36.725***	43.311***	32.009***	39.330***	41.247***	38.236***	31.497***	37.440***
	(1.31)	(1.36)	(1.21)	(1.26)	(1.45)	(1.19)	(1.20)	(1.21)
								
p-value of coefficient on Top Quartile X Age	0	0.536	0.023	0	0.01	0.743	0.697	0
								
N	2048	2244	2314	2241	2012	2340	2271	2415

N= number of children in top & bottom quartiles x number of rounds. Some children are missing outcomes in intermediate rounds.

Significant at *0.1 **0.05 ***0.01.

Standard errors clustered by child.

a Andhra Pradesh and Telangana.

**Fig. A4:**
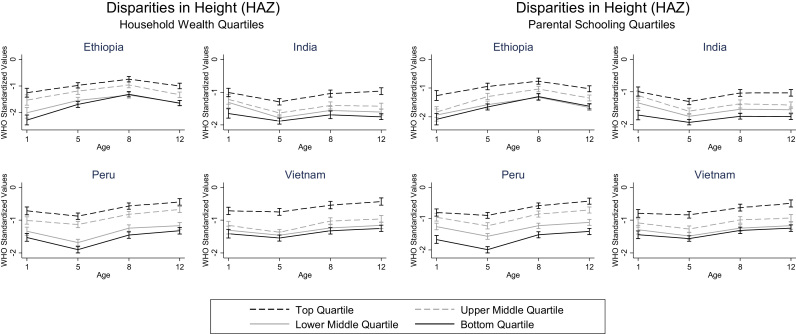
Disparities in HAZ from household wealth and parental schooling are fairly parallel, Young Lives study. HAZ is standardized according to World Health Organization international norms.

A supplementary analysis indicates similar findings when height is represented as height-for-age z-score, with a few key differences (Fig. A4). In Ethiopia, the disparities in percentile height are parallel, but the disparities in HAZ shrink as children age. In India, the disparities in percentile height widen with age, but the disparities in HAZ are parallel (Table A5). We perform a robustness check for Ethiopia using the smaller sample with only speakers of Amharic, the majority language in the sample (Table A6). Within this population, for all disparities assessed we do not reject the hypothesis of parallel trends. We confirm that parallelism emerges from the quartiles’ mean scores staying constant rather than both increasing or—more concerning—worsening over time: by testing that the lowest quartiles’ linear trends differ from zero, we find only two cases of decline with respect to wealth (PPVT for Ethiopia and India) and none with respect to parental schooling.

We do not find significant differences in vocabulary based on puberty marker status. Observations with data on absence or presence of puberty markers range from 93% (boys in Ethiopia) to 100% (girls in Peru). Adjusting for puberty status reduces the height gap at age 12 between 7% (Peru, parental schooling disparity) and 55% (India, parental schooling disparity), except in Ethiopia, where the low prevalence of puberty markers by age 12 (9%) makes this adjustment specious. In most cases, the fraction of children with puberty markers is higher in the top SES quartiles than the bottom SES quartiles (Table A7). These exceptions (males in Ethiopia & India) are found where small fractions of children exhibit puberty markers. Without exception, girls and boys with puberty makers are taller than children without puberty markers.

**Fig. 2:**
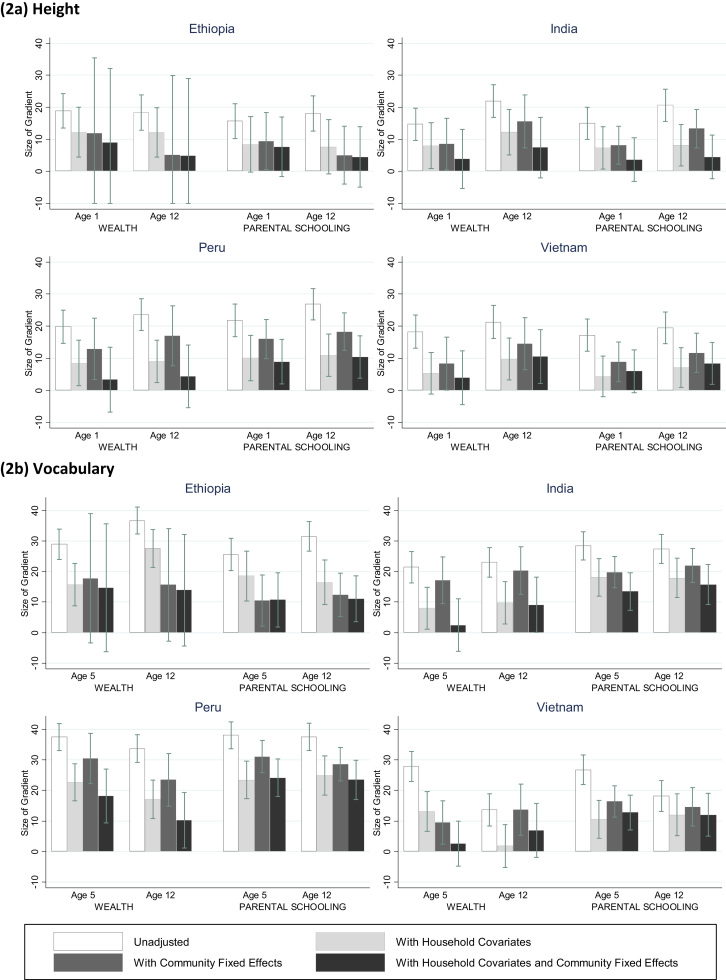
Disparities in height and vocabulary from wealth and parental schooling at ages 1 and 12 y, with and without controls for household variables and community fixed effects, Young Lives study. Household covariates are mother’s height in centimeters, mother’s age in years, ethnicity indicator variables, and an indicator variable for the mother speaking the region’s official language. The variable not defining the disparitiy is also included: the parental schooling index is included as a household covariate in the adjusted disparities from wealth and vice versa.). Community fixed effects are defined by location at age 1y. Confidence intervals are wide for community fixed effects in Ethiopia for the wealth disparity because 14 of 20 communities do not contain households in both top and bottom quartile. Confidence intervals for Ethiopian wealth disparities adjusted for community fixed effects and mother characteristics (height, age, ethnicity, and language) with community fixed effects are truncated at −10. Vocabulary (PPVT) is standardized within country and language of administration.

**Fig. A5:**
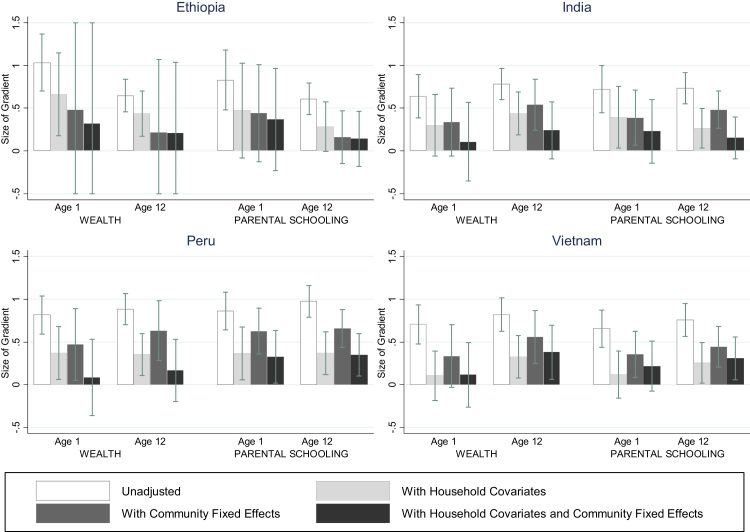
Disparities in HAZ from wealth and parental schooling at ages 1 and 12 y, with and without controls for household variables and community fixed effects, Young Lives study. Household covariates are mother’s height in centimeters, mother’s age in years, ethnicity indicator variables, and an indicator variable for the mother speaking the region’s official language. The variable not defining the disparitiy is also included: the parental schooling index is included as a household covariate in the adjusted disparities from wealth and vice versa.). Community fixed effects are defined by location at age 1y. Confidence intervals are wide for community fixed effects in Ethiopia for the wealth disparity because 14 of 20 communities do not contain households in both top and bottom quartile. Confidence intervals for Ethiopian wealth disparities adjusted for community fixed effects and mother characteristics (height, age, ethnicity, and language) with community fixed effects are truncated at −10 for height and vocabulary and are truncated at −0.5 and 1.5. HAZ is standardized according to World Health Organization Growth Standards/References (de Onis et al., 2007; World Health Organization, 2006).

For most of the analyses, the disparity in height is no longer statistically significant when adjusting for the combination of mother characteristics and community fixed effects (Fig. 2a and Table A8). Household variables generally are more strongly associated with the disparities in height than community fixed effects, but these are not statistically distinct. The supplementary analysis indicates similar findings when height is represented as height-for-age z-score HAZ (Fig. A5). In contrast, the gaps in percentile vocabulary from wealth remain statistically significant in many cases when adjusting for household characteristics and community fixed effects; the adjusted gap is around 50% of the size of the unadjusted gap (Fig. 2b and Table A8). In two cases (Ethiopia and Vietnam for vocabulary) disparities at first age measured and at 12y are significantly different. Yet when we include maternal characteristics and community fixed effects, the adjusted disparities at age first measured and at 12y are of a similar magnitude.

## Discussion

We find that children living in households with more material resources and/or with parents who have more schooling are taller and perform better on a test of receptive vocabulary on average than children who live with less wealth or with parents who have attained fewer grades of schooling; these findings are consistent across four low- or middle-income countries at all ages measured. The differences in mean percentile height between children living in low and high SES households are established early in life and persist —and in a few cases they increase—between 1y and 12y. In contrast, disparities in vocabulary do not have consistent trends across ages from when they were first measured (5y) through to the final measurement (12y). The SES gaps in percentile vocabulary are larger in general than SES gaps in percentile height.

In contrast to our hypotheses, our results indicate that disparities in vocabulary between the first and fourth SES quartile on average may increase or decrease over time, depending on the context. This finding implies that policies may be able to influence vocabulary, which is a conclusion supported by the understanding of sensitive periods in the science of development in early and middle childhood (Adair, 1999, Behrman, 2015, Crookston et al., 2010, Crookston et al., 2011, Crookston et al., 2013, Georgiadis et al., 2016, Lundeen et al., 2014, Mani, 2012, Penny et al., 2015, Prentice et al., 2013, Schott et al., 2013). Similarly, these findings support the understanding that cognition can be plastic for a longer period of time than linear growth. This result contrasts with the existing research in lower- and middle-income countries that suggests disparities in vocabulary stabilize during middle childhood (Galasso et al., 2017, Schady et al., 2015).

Disparities in height at first measurement and at 12y are of similar magnitude when adjusted for household covariates at age 1y and community fixed effects, suggesting that early life environments greatly influence childhood trajectories. Since both household-level variables and community fixed effects reduce coefficients for the disparities in height and language, exposures at both the micro- and macro-levels influence childhood inequality early on in life. Conditions associated with poverty, such as low birth weight, stress-induced hormonal changes during pregnancy, and postnatal exposure to environmental stressors, are possible mechanisms for variations in development (Boyce and Ellis, 2005, Hertzman and Boyce, 2010). For these reasons, policy makers who prioritize equitable childhood development outcomes should integrate education and stimulation into early childhood interventions. Successful interventions are often implemented as multi-sectoral packages anchored in nurturing care and include parenting support, preschool participation, and responsive care, among others (Britto et al., 2016).

Disparities in vocabulary from SES were generally larger than disparities in height from SES, and were not fully explained by household covariates or community fixed effects. Patterns by which these covariates attenuated the height and vocabulary gaps were similar across countries. There are several mechanisms that connect household wealth and parental education with childhood growth and cognitive outcomes. A household with more wealth is more likely to purchase and provide adequate and nourishing food or cognitive stimulation for children (Black et al., 2016). Formal education may equip parents to successfully apply knowledge about health, sanitation, and responsive interaction when caring for their children (Fuchs, Pamuk, & Lutz, 2010). Interventions to improve family SES have proven helpful in improving child outcomes (Britto et al., 2016). Future research could explore a pathway analysis to understand the role of each variable (Prado et al., 2017) as well as considering how the outcomes influence each other; nutrition has been found to influence cognition in these populations (Georgiadis et al., 2016).

In spite of the strengths of our study, there are also some clear limitations. The data are not nationally-representative but over-represent the poor, and our analytic sample is generally wealthier than those omitted from the overall sample, likely due to exclusion of children who took the vocabulary test in one of the minority languages. Thus our estimates may be conservative. Our study is descriptive, with no effort to identify causal effects. Our measure of parental years of schooling is only available at child age 5y, so we may be mis-categorizing any parents who acquired schooling since child age 1y, though this is at most 2.5% of each country sample. We have data from a longer time period for height than for vocabulary, so we cannot comment on very early SES gaps in cognition. Though we examine height differences by puberty markers, this sensitivity analysis remains speculative, but it suggests that our main estimates of height disparities may be reduced by 23%, on average, after puberty growth. There is a large range of variables that were not collected at age 1y, such as parental stimulation, school quality, home environment, and consumption of nutritious foods, all of which are likely to be on the causal pathways connecting SES and health/development outcomes. We lack information on social or family violence and access to early childhood education, which may also influence cognitive stimulation and may explain disparities in vocabulary. Thus, we are limited in our ability to comment much on mechanistic pathways. Finally we focus on the average values for the first and fourth baseline SES quartiles, though there may be movement within these quartiles with some children improving and others faltering that is masked by the quartile averages.

In this study, we add to the existing literature by assessing disparities in height and vocabulary from SES across an extended period of childhood in four developing countries. The Young Lives study country samples represent the diversity of children in each context and provide an invaluable opportunity for examining these SES gaps in resource-poor settings. Our findings suggest that policy changes through interventions early in life to improve inequality can have large and generally persistent impacts, since basic patterns in SES gaps are established early, as supported by a large body of literature (Engle et al., 2007, Engle et al., 2011, Grantham-McGregor et al., 2007, Hoddinott et al., 2013b, Hoddinott et al., 2013a, Hoddinott et al., 2008, Maluccio et al., 2009, Martorell et al., 2010, Victora et al., 2008, Victora et al., 2010). Our study raises questions for further research. Longitudinal studies with data on cognitive development early in life are necessary to better document how and when vocabulary disparities emerge. Cultural, political, and historical research could help explain differences across contexts. While our data cover a long time span, they do not include adolescence and adulthood. More information about the transitions to adulthood will be necessary to determine if these disparities persist.

## Figures and Tables

**Fig. A2:**
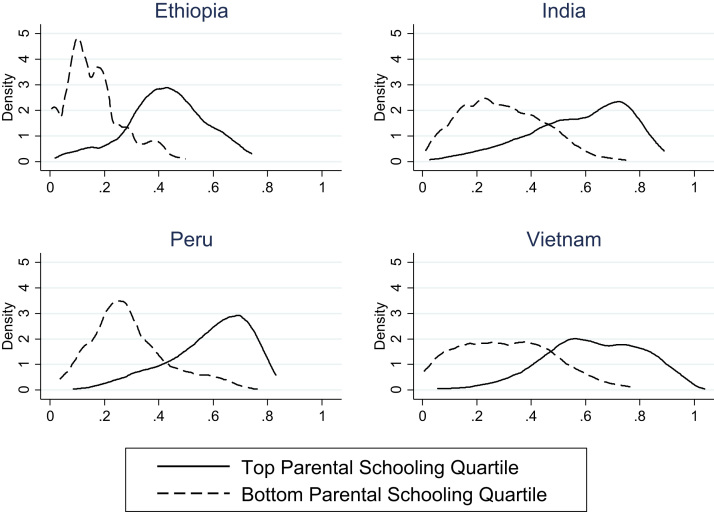
Densities of Wealth Index by Average Parental Schooling.

**Fig. A3:**
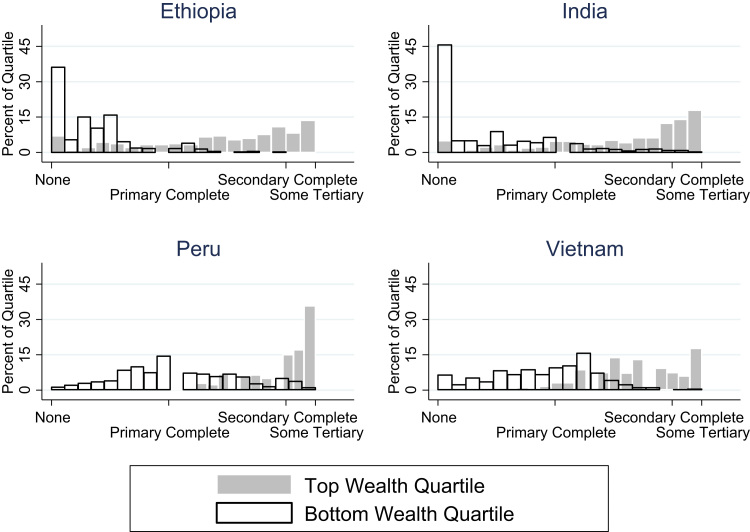
Densities of Average Parental Schooling by Wealth Quartile.

**Table A1:** Defining Analytic Sample.

	Original sample - N at age 1y		Analytic Sample	Analytic sample as a percentage of original sample	N in top or bottom quartile Wealth	N in top or bottom quartile Parental Schooling
Data on height and vocabulary in first & last rounds^a^		x	x			
PPVT in major languages first & last rounds			x			
Ethiopia	1986	1764	1345	68%	687	676
India (AP & TG)^b^	2011	1793	1604	80%	800	836
Peru	2052	1779	1574	77%	787	776
Vietnam	2000	1628	1514	76%	766	823

First rounds are age 1 year for HAZ and age 5 years for age and language standardized PPVT.

Last rounds are age 12 years for both HAZ and age and language standardized PPVT

In Ethiopia, 1999 children were surveyed, but only 1986 had wealth data. All children in other countries had complete wealth data.

Information on parental education was available for all children.

a Children not included if HAZ is beyond 6 S.D.

b Andhra Pradesh and Telangana

**Table A2:** In-analytic-sample and out-of-analytic-sample means.

		**Ethiopia**		**India (AP & TG)**^b^		**Peru**		**Vietnam**	
	**Variables**	in sample	out of sample	in sample	out of sample	in sample	out of sample	in sample	out of sample
Disparities	Wealth Index	0.24	0.15***	0.40	0.42	0.45	0.33***	0.46	0.39***
	Average Parental Schooling Level^a^	2.75	2.43	3.51	3.39	6.19	4.42***	5.22	4.1***
									
HAZ	Height-for-age z-score age 1 (adjusted)	-1.78	-1.91	-1.30	-1.30	-1.15	-1.91***	-1.15	-1.37***
	Height-for-age z-score age 5	-1.36	-1.67***	-1.65	-1.62	-1.40	-2.1***	-1.28	-1.53***
	Height-for-age z-score age 8	-1.09	-1.48***	-1.43	-1.45	-1.03	-1.62***	-1.03	-1.31***
	Height-for-age z-score age 12	-1.41	-1.6***	-1.44	-1.47	-0.90	-1.52***	-0.95	-1.32***
									
Mother Variables	Mother Speaks Local Language Fluently	0.82	0.86	0.43	0.35***	0.11	0.06	0.96	0.72***
	Mother's Age	27.41	27.26	23.57	24.10	26.62	27.35	27.21	27.07
	Mother's Height (cm)	158.77	158.63	151.54	151.81	150.23	148.96***	152.44	151.32***
									
Community Variables	Average Community Wealth Index (Excluding the child's)	0.24	0.15***	0.40	0.43***	0.45	0.35***	0.45	0.41***
	Rural	0.56	0.83***	0.77	0.66***	0.25	0.63***	0.81	0.78
	Community has Health Clinic or Hospital within 10K	0.87	0.83	0.53	0.52	0.69	0.72	0.63	0.54***
	Community has Secondary School or Higher within 10K	0.41	0.52***	0.38	0.40	0.60	0.59	0.98	0.86***
								
	N	1345	654	1604	407	1574	478	1514	486

Difference between means is statistically significant *p=0.1 **p=0.05 ***p=0.01

PPVT summary statistics are not presented since language of the PPVT is the main limiter of being included in the sample

Missing values are imputed

a Levels are from 0 - 8: (0) no education (1) incomeplete lower primary (2) complete lower primary….(8) complete upper secondary.

b Andhra Pradesh and Telangana

**Table A3:** Top & bottom wealth quartiles' mean values for components of the wealth index at 1y.

		**Ethiopia**		**India (AP & TG)**^b^		**Peru**		**Vietnam**	
	**Variables**	**Top Quartile**	**Bottom Quartile**	**Top Quartile**	**Bottom Quartile**	**Top Quartile**	**Bottom Quartile**	**Top Quartile**	**Bottom Quartile**
Housing Quality	Crowding (rooms per person)	0.38	0.21	0.45	0.28	0.58	0.37	0.52	0.33
	Wall material satisfies quality norms	0.16	0.15	0.96	0.07	0.92	0.00	0.99	0.07
	Roof material satisfies quality norms	0.97	0.02	0.88	0.20	0.97	0.72	0.99	0.30
	Floor material satisfies quality norms	0.44	0.00	0.56	0.02	0.97	0.00	0.93	0.13
									
Consumer Durables^a^	Radio	0.83	0.07	0.32	0.12	0.90	0.59	0.64	0.36
	Refrigerator	0.03	0.00	0.17	0.01	0.56	0.00	0.35	0.01
	Bicycle	0.10	0.00	0.51	0.14	0.45	0.16	0.76	0.59
	Television	0.27	0.00	0.72	0.03	0.96	0.17	0.90	0.28
	Motorbicycle	0.00	0.00	0.30	0.00	0.07	0.00	0.85	0.16
	Car	0.02	0.00	0.01	0.00	0.15	0.00	0.03	0.00
	Mobile Phone	0.01	0.00	0.04	0.00	0.21	0.00	0.22	0.00
	Landline Phone	0.17	0.00	0.22	0.00	0.33	0.00	0.45	0.00
									
Services	Access to safe drinking water	0.93	0.27	0.96	0.73	0.87	0.15	0.37	0.00
	Access to sanitation	0.51	0.02	0.83	0.01	0.99	0.54	0.93	0.10
	Access to electricity	0.98	0.00	0.99	0.47	1.00	0.11	1.00	0.59
	Access to adequate fuels for cooking	0.37	0.00	0.77	0.00	0.95	0.06	0.73	0.02
								
	N	336	351	397	403	386	401	378	388

Difference between means is statistically significant *p=0.1 **p=0.05 ***p=0.01

The subset of variables which are present in all countries' wealth index was selected to provide better conceptualization of wealth.

a Not all consumer durables listed; others different across countries.

b Andhra Pradesh and Telangana.

**Table A4:** Percentage of missing data in the top and bottom SES quartiles.

		**Ethiopia**		**India (AP & TG)**^a^		**Peru**		**Vietnam**	
		**Wealth**	**Parental Schooling**	**Wealth**	**Parental Schooling**	**Wealth**	**Parental Schooling**	**Wealth**	**Parental Schooling**
	**Variables**								
Disparities	Wealth Index	0%	0%	0%	0%	0%	0%	0%	0%
	Average Parental Schooling	0%	0%	0%	0%	0%	0%	0%	0%
									
Standardized Outcomes	Age & language standardized PPVT score age 5	0%	0%	0%	0%	0%	0%	0%	0%
	Age & language standardized PPVT score age 8	2%	2%	19%	20%	6%	7%	7%	7%
	Age & language standardized PPVT score age 12	0%	0%	0%	0%	0%	0%	0%	0%
	Height-for-age z-score age 1 (adjusted)	0%	0%	0%	0%	0%	0%	0%	0%
	Height-for-age z-score age 5	0%	0%	0%	0%	0%	0%	0%	0%
	Height-for-age z-score age 8	1%	1%	0%	0%	1%	1%	1%	1%
	Height-for-age z-score age 12	0%	0%	0%	0%	0%	0%	0%	0%
									
Mother Variables	Mother Speaks Local Language Fluently	1%	0%	0%	0%	0%	0%	0%	0%
	Mother's Age	4%	4%	1%	1%	1%	1%	0%	0%
	Mother's Ethnicity	0%	0%	0%	0%	0%	0%	0%	0%
	Mother's Height (cm)	10%	11%	2%	3%	5%	4%	1%	0%
								
	N	687	676	800	836	787	776	766	823

a Andhra Pradesh and Telangana

**Table A5:** SES Disparities in HAZ between Top and Bottom Quartiles & Testing for Parallel Trends.

	**Wealth Disparity**				**Schooling Disparity**			
	**Ethiopia**	**India (AP & TG)**^a^	**Peru**	**Vietnam**	**Ethiopia**	**India (AP & TG)**^a^	**Peru**	**Vietnam**
**HAZ - WHO stand/ref: Ages 1, 5, 8, & 12**	Coef./SE	Coef./SE	Coef./SE	Coef./SE	Coef./SE	Coef./SE	Coef./SE	Coef./SE
Top Quartile Indicator Variable	0.980***	0.578***	0.888***	0.714***	0.817***	0.687***	0.928***	0.653***
(SES Gap at Age 1)	(0.11)	(0.09)	(0.08)	(0.09)	(0.12)	(0.10)	(0.08)	(0.08)
								
Age 5 Indicator Variable	0.504***	-0.287***	-0.275***	-0.100***	0.420***	-0.269***	-0.218***	-0.099***
	(0.07)	(0.05)	(0.04)	(0.03)	(0.07)	(0.05)	(0.04)	(0.03)
								
Age 8 Indicator Variable	0.872***	-0.085	0.101**	0.100**	0.725***	-0.05	0.164***	0.126***
	(0.08)	(0.06)	(0.04)	(0.05)	(0.07)	(0.06)	(0.04)	(0.04)
								
Age 12 Indicator Variable	0.647***	-0.105	0.226***	0.172***	0.469***	-0.057	0.278***	0.206***
	(0.09)	(0.07)	(0.05)	(0.06)	(0.09)	(0.07)	(0.05)	(0.05)
								
**Top Quartile (Indicator) X Age (Continuous)**	-0.036***	**0.013**	**0.003**	**0.01**	-0.022**	**0.003**	**0.006**	**0.008**
	**(0.01)**	**(0.01)**	**(0.01)**	**(0.01)**	**(0.01)**	**(0.01)**	**(0.01)**	**(0.01)**
								
Constant (Bottom Quartile at Age 1)	-2.245***	-1.624***	-1.568***	-1.427***	-2.073***	-1.697***	-1.700***	-1.448***
	(0.09)	(0.07)	(0.06)	(0.06)	(0.09)	(0.07)	(0.06)	(0.06)
								
p-value of coefficient on Top Quartile X Age	0.001	0.101	0.72	0.148	0.036	0.753	0.399	0.214
								
N	2744	3197	3138	3053	2699	3341	3092	3284

N= number of children in top & bottom quartiles x number of rounds. Some children are missing outcomes in intermediate rounds

Significant at *0.1 **0.05 ***0.01

Standard errors clustered by child

a Andhra Pradesh and Telangana

**Table A6:** Robustness check for Ethiopia of SES disparities between top and bottom quartiles & testing for parallel trends.

	**Wealth Disparity**	**Schooling Disparity**
	**Ethiopia**	**Ethiopia**
Panel A		
**Percentile Height: Ages 1, 5, 8, & 12**	Coef./SE	Coef./SE
Top Quartile Indicator Variable	19.67***	20.53***
(SES Gap at Age 1)	(2.78)	(2.80)
		
Age 5 Indicator Variable	0.4	0.41
	(1.62)	(1.74)
		
Age 8 Indicator Variable	-1.18	1.15
	(1.83)	(1.91)
		
Age 12 Indicator Variable	-2.34	0.57
	(2.29)	(2.35)
		
**Top Quartile (Indicator) X Age (Continuous)**	**0.35**	**-0.11**
	**(0.29)**	**(0.29)**
		
Constant (Bottom Quartile at Age 1)	40.04***	38.91***
	(1.96)	(2.07)
		
p-value of coefficient on Top Quartile X Age	0.24	0.701
		
N	1474	1299

	**Wealth Disparity**	**Schooling Disparity**
	**Ethiopia**	**Ethiopia**
Panel B		
**Percentile Vocabulary: Ages 5, 8, & 12**	Coef./SE	Coef./SE
Top Quartile Indicator Variable	40.93***	36.36***
(SES Gap at Age 5)	(3.32)	(3.89)
		
Age 8 Indicator Variable	-2.66*	-0.16
	(1.51)	(1.78)
		
Age 12 Indicator Variable	-5.27***	-0.57
	(1.65)	(2.21)
		
**Top Quartile (Indicator) X Age (Continuous)**	**0.52**	**0.23**
	**(0.36)**	**(0.43)**
		
Constant (Bottom Wealth Quartile at Age 5)	27.72***	31.63***
	(1.27)	(1.68)
		
p-value of coefficient on Top Quartile X Age	0.152	0.592
N	1101	970

	**Wealth Disparity**	**Schooling Disparity**
	**Ethiopia**	**Ethiopia**
Panel C		
**HAZ - WHO stand/ref: Ages 1, 5, 8, & 12**	Coef./SE	Coef./SE
Top Quartile Indicator Variable	0.96***	0.99***
(SES Gap at Age 1)	(0.14)	(0.16)
Age 5 Indicator Variable	0.60***	0.57***
	(0.09)	(0.10)
		
Age 8 Indicator Variable	0.82***	0.86***
	(0.10)	(0.11)
		
Age 12 Indicator Variable	0.52***	0.60***
	(0.12)	(0.13)
		
**Top Quartile (Indicator) X Age (Continuous)**	**-0.02**	-0.03**
	**(0.01)**	**(0.02)**
		
Constant (Bottom Quartile at Age 1)	-2.24***	-2.24***
	(0.11)	(0.12)
		
p-value of coefficient on Top Quartile X Age	0.227	0.025
		
N	1474	1299

N= number of children in top & bottom quartiles x number of rounds. Some children are missing outcomes in intermediate rounds.

Significant at *0.1 **0.05 ***0.01.

Standard errors clustered by child.

**Table A7:** 

**Panel A: Mean Height Percentile by Quartile & Puberty Marker**																			
	**Ethiopia**					**India - Andhra Pradesh and Telangana**					**Peru**					**Vietnam**			
	**Does not Menstruate**	**Menstruates**	**Voice has not lowered**	**Low voice**		**Does not Menstruate**	**Menstruates**	**Voice has not lowered**	**Low voice**		**Does not Menstruate**	**Menstruates**	**Voice has not lowered**	**Low voice**		**Does not Menstruate**	**Menstruates**	**Voice has not lowered**	**Low voice**
**Wealth**	**Girls (N=307)**		**Boys (N=377)**			**Girls (N=371)**		**Boys (N=429)**			**Girls (N=386)**		**Boys (N=401)**			**Girls (N=388)**		**Boys (N=381)**	
Bottom Quartile	40.71	50.00	44.04	58.48		35.85	67.29	41.39	44.90		34.64	56.67	37.84	40.43		40.16	58.80	38.12	52.74
N	151	2	156	25		159	31	189	21		158	46	102	94		173	35	134	42
Top Quartile	64.10	77.50	60.73	52.50		53.88	78.89	64.88	68.33		56.20	72.61	62.64	63.51		50.00	70.48	59.27	74.71
N	143	6	160	10		125	53	196	18		111	71	89	115		75	95	105	93
Adjustments by sex		3.69		-20.19			-8.75		0.00			-7.05		-0.97			-6.47		-2.94
Gradient adjustment				-44%					-19%					-18%					-22%
																			
**Parents' Education**	**Does not Menstruate**	**Menstruates**	**Voice has not lowered**	**Low voice**		**Does not Menstruate**	**Menstruates**	**Voice has not lowered**	**Low voice**		**Does not Menstruate**	**Menstruates**	**Voice has not lowered**	**Low voice**		**Does not Menstruate**	**Menstruates**	**Voice has not lowered**	**Low voice**
	**Girls (N=303)**		**Boys (N=374)**			**Girls (N=365)**		**Boys (N=471)**			**Girls (N=377)**		**Boys (N=399)**			**Girls (N=400)**		**Boys (N=425)**	
Bottom Quartile	42.23	47.00	44.16	53.50		36.36	68.71	40.50	50.25		31.06	48.74	35.27	40.59		39.86	65.42	35.47	52.48
N	151	1	173	10		160	24	214	28		139	47	96	88		172	36	136	69
Top Quartile	63.90	83.60	59.75	57.38		55.41	75.89	60.72	60.18		56.53	75.05	61.08	64.93		52.81	69.32	56.69	74.58
N	140	5	157	13		112	61	202	22		116	75	93	121		106	81	114	95
Adjustments by sex		14.28		-11.02			-14.87		-9.11			-1.96		-1.10			-11.78		-1.53
Gradient adjustment				1%					-55%					-7%					-31%
													

**Panel B: Mean HAZ by Quartile & Puberty Marker**																			
	**Ethiopia**					**India - Andhra Pradesh and Telangana**					**Peru**					**Vietnam**			
	**Does not Menstruate**	**Menstruates**	**Voice has not lowered**	**Low voice**		**Does not Menstruate**	**Menstruates**	**Voice has not lowered**	**Low voice**		**Does not Menstruate**	**Menstruates**	**Voice has not lowered**	**Low voice**		**Does not Menstruate**	**Menstruates**	**Voice has not lowered**	**Low voice**
**Wealth**	**Girls (N=307)**		**Boys (N=377)**			**Girls (N=371)**		**Boys (N=429)**			**Girls (N=386)**		**Boys (N=401)**			**Girls (N=388)**		**Boys (N=381)**	
Bottom Quartile	-1.77	-1.42	-1.63	-1.15		-1.97	-0.88	-1.74	-1.59		-1.51	-0.67	-1.35	-1.29		-1.34	-0.68	-1.40	-0.84
N	151	2	156	25		159	31	189	21		158	46	102	94		173	35	134	42
Top Quartile	-0.90	-0.10	-1.09	-1.40		-1.35	-0.37	-0.91	-0.85		-0.73	-0.07	-0.47	-0.38		-0.96	-0.27	-0.60	0.07
N	143	6	160	10		125	53	196	18		111	71	89	115		75	95	105	93
Adjustments by sex		0.42		-0.71			-0.22		-0.08			-0.25		0.01			-0.24		-0.07
Gradient adjustment				-25%					-18%					-15%					-20%
																			
**Parents' Education**	**Does not Menstruate**	**Menstruates**	**Voice has not lowered**	**Low voice**		**Does not Menstruate**	**Menstruates**	**Voice has not lowered**	**Low voice**		**Does not Menstruate**	**Menstruates**	**Voice has not lowered**	**Low voice**		**Does not Menstruate**	**Menstruates**	**Voice has not lowered**	**Low voice**
	**Girls (N=303)**		**Boys (N=374)**			**Girls (N=365)**		**Boys (N=471)**			**Girls (N=377)**		**Boys (N=399)**			**Girls (N=400)**		**Boys (N=425)**	
Bottom Quartile	-1.68	-1.5	-1.62	-1.35		-1.97	-0.85	-1.75	-1.45		-1.63	-0.95	-1.44	-1.27		-1.37	-0.39	-1.5	-0.89
N	151	1	173	10		160	24	214	28		139	47	96	88		172	36	136	69
Top Quartile	-0.96	0.17	-1.1	-1.24		-1.29	-0.51	-1.05	-1.15		-0.72	0.01	-0.53	-0.37		-0.87	-0.32	-0.7	0.08
N	140	5	157	13		112	61	202	22		116	75	93	121		106	81	114	95
Adjustments by sex		0.91		-0.38			-0.47		-0.36			-0.06		-0.02			-0.5		0.02
Gradient adjustment				26%					-53%					-5%					-30%

Adjustments by sex: for each quartile, the difference between the average percentile heights of those who have entered and those who have not entered puberty is calculated. This difference is multipled by the fraction of girls in the quartile who have not yet reached puberty, indicating how much growth we may expect to be added to the mean of the quartile. Then we take the difference between expected growth of the top and bottom quartiles.

The gradient adjustment: the average of the adjustments by sex weighted proportionally by boys and girls in the analytical sample, and divided by the total size of the gradient.

Note: some children are missing data on these puberty outcomes. We use weights based on all children in the analytical sample, not just those with puberty indicator data.

**Table A8:** Disparities & Adjusted Disparities with 99% Confidence Intervals.

	Disparities at first measurement*				**Disparities at age 12y**					Disparities at first measurement*				**Disparities at age 12y**			
	**Ethiopia**									**Ethiopia**							
	**Unadjusted**	**Household Covariates**	**Community FE**	**Household + Comm. FE**	**Unadjusted**	**Household Covariates**	**Community FE**	**Household + Comm. FE**		**Unadjusted**	**Household Covariates**	**Community FE**	**Household + Comm. FE**	**Unadjusted**	**Household Covariates**	**Community FE**	**Household + Comm. FE**
**Height (percentile)**									**Height (HAZ)**								
																	
Wealth disparity	18.8***	12.2***	11.9	8.9	18.5***	12.4***	5.2	4.8	Wealth disparity	0.8***	0.5**	0.4**	0.4	0.6***	0.3**	0.2	0.1
	[13.4,24.2]	[4.4,19.9]	[-11.6,35.5]	[-14.3,32.2]	[13.0,24.0]	[4.7,20.1]	[-19.5,29.9]	[-19.2,28.9]		[0.5,1.2]	[-0.1,1.0]	[-0.1,1.0]	[-0.2,1.0]	[0.4,0.8]	[-0.0,0.6]	[-0.1,0.5]	[-0.2,0.5]
**Height (percentile)**									**Height (HAZ)**								
																	
Schooling disparity	15.6***	8.5**	9.4***	7.6**	18.0***	7.7**	5	4.5	Schooling disparity	1.0***	0.7***	0.5	0.3	0.7***	0.4***	0.2	0.2
	[10.1,21.1]	[-0.2,17.1]	[0.6,18.3]	[-1.7,16.9]	[12.6,23.5]	[-0.8,16.2]	[-4.0,14.0]	[-5.0,13.9]		[0.7,1.4]	[0.2,1.1]	[-1.0,2.0]	[-1.2,1.8]	[0.5,0.8]	[0.2,0.7]	[-0.6,1.1]	[-0.6,1.0]
																
**Vocabulary (percentile)**																	
																	
Wealth disparity	25.5***	18.5***	10.5***	10.7***	31.4***	16.5***	12.4***	11.1***									
	[20.2,30.7]	[10.4,26.7]	[2.1,18.8]	[1.7,19.6]	[26.6,36.2]	[9.2,23.8]	[5.3,19.4]	[3.6,18.5]									
																
**Vocabulary (percentile)**																	
																	
Schooling disparity	29.0***	15.9***	17.9**	14.8*	36.7***	27.6***	15.6**	13.9**									
	[24.1,33.9]	[9.0,22.8]	[-3.3,39.0]	[-6.2,35.7]	[32.3,41.0]	[21.4,33.7]	[-2.8,34.0]	[-4.3,32.1]									
			

	**India - Andhra Pradesh and Telangana**									**India - Andhra Pradesh and Telangana**							
																
	**Unadjusted**	**Household Covariates**	**Community FE**	**Household + Comm. FE**	**Unadjusted**	**Household Covariates**	**Community FE**	**Household + Comm. FE**		**Unadjusted**	**Household Covariates**	**Community FE**	**Household + Comm. FE**	**Unadjusted**	**Household Covariates**	**Community FE**	**Household + Comm. FE**
**Height (percentile)**									**Height (HAZ)**								
																	
Wealth disparity	14.7***	8.0***	8.5***	3.9	21.9***	12.3***	15.6***	7.4**	Wealth disparity	0.6***	0.3**	0.3**	0.1	0.8***	0.4***	0.5***	0.2*
	[9.7,19.8]	[0.8,15.2]	[0.5,16.5]	[-5.4,13.2]	[16.9,27.0]	[5.2,19.4]	[7.3,23.9]	[-2.0,16.8]		[0.4,0.9]	[-0.1,0.7]	[-0.1,0.7]	[-0.4,0.6]	[0.6,1.0]	[0.2,0.7]	[0.2,0.8]	[-0.1,0.6]
**Height (percentile)**									**Height (HAZ)**								
																	
Schooling disparity	15.0***	7.3***	8.2***	3.7	20.7***	8.1***	13.3***	4.5*	Schooling disparity	0.7***	0.4***	0.4***	0.2	0.7***	0.3***	0.5***	0.2
	[10.0,20.0]	[0.7,14.0]	[2.2,14.1]	[-3.1,10.5]	[15.7,25.7]	[1.7,14.6]	[7.3,19.4]	[-2.3,11.4]		[0.4,1.0]	[0.0,0.8]	[0.1,0.7]	[-0.1,0.6]	[0.6,0.9]	[0.0,0.5]	[0.3,0.7]	[-0.1,0.4]
																
**Vocabulary (percentile)**																	
																	
Wealth disparity	21.4***	8.0***	17.1***	2.5	23.0***	9.8***	20.3***	9.1***									
	[16.3,26.5]	[1.1,14.9]	[9.6,24.7]	[-6.1,11.1]	[18.2,27.9]	[2.9,16.7]	[12.6,28.1]	[0.1,18.1]									
																
**Vocabulary (percentile)**																	
																	
Schooling disparity	28.4***	18.1***	19.8***	13.5***	27.4***	17.9***	22.0***	15.8***									
	[23.8,33.1]	[12.0,24.2]	[14.6,25.0]	[7.3,19.6]	[22.7,32.1]	[11.5,24.3]	[16.4,27.5]	[9.2,22.4]									

	Disparities at first measurement*				**Disparities at age 12y**				Disparities at first measurement*					**Disparities at age 12y**			
	**Peru**									**Peru**							
	**Unadjusted**	**Household Covariates**	**Community FE**	**Household + Comm. FE**	**Unadjusted**	**Household Covariates**	**Community FE**	**Household + Comm. FE**		**Unadjusted**	**Household Covariates**	**Community FE**	**Household + Comm. FE**	**Unadjusted**	**Household Covariates**	**Community FE**	**Household + Comm. FE**
**Height (percentile)**									**Height (HAZ)**								
																	
Wealth disparity	19.8***	8.5***	12.9***	3.3	23.6***	9.0***	17.0***	4.4	Wealth disparity	0.8***	0.4***	0.5***	0.1	0.9***	0.4***	0.6***	0.2
	[14.7,25.0]	[1.4,15.6]	[3.4,22.5]	[-6.8,13.4]	[18.7,28.5]	[2.5,15.6]	[7.7,26.3]	[-5.4,14.1]		[0.6,1.0]	[0.1,0.7]	[0.1,0.9]	[-0.4,0.5]	[0.7,1.1]	[0.1,0.6]	[0.3,1.0]	[-0.2,0.5]
**Height (percentile)**									**Height (HAZ)**								
																	
Schooling disparity	21.8***	10.1***	16.0***	8.9***	26.8***	10.9***	18.3***	10.4***	Schooling disparity	0.9***	0.4***	0.6***	0.3***	1.0***	0.4***	0.7***	0.3***
	[16.7,26.8]	[3.0,17.2]	[9.9,22.1]	[2.0,15.8]	[22.0,31.7]	[4.3,17.6]	[12.5,24.1]	[3.8,17.0]		[0.6,1.1]	[0.1,0.7]	[0.4,0.9]	[0.0,0.6]	[0.8,1.2]	[0.1,0.6]	[0.4,0.9]	[0.1,0.6]
																
**Vocabulary (percentile)**																	
																	
Wealth disparity	37.5***	22.6***	30.4***	18.2***	33.7***	17.1***	23.4***	10.2***									
	[33.1,41.9]	[16.6,28.6]	[22.2,38.7]	[9.4,27.0]	[29.1,38.2]	[10.9,23.3]	[14.9,32.0]	[1.2,19.3]									
																
**Vocabulary (percentile)**																	
																	
Schooling disparity	38.0***	23.4***	31.0***	24.1***	37.5***	24.9***	28.6***	23.4***									
	[33.6,42.4]	[17.3,29.6]	[25.8,36.3]	[18.0,30.3]	[33.0,42.0]	[18.5,31.3]	[23.1,34.1]	[17.0,29.9]									
			

	**Vietnam**									**Vietnam**							
																
	**Unadjusted**	**Household Covariates**	**Community FE**	**Household + Comm. FE**	**Unadjusted**	**Household Covariates**	**Community FE**	**Household + Comm. FE**		**Unadjusted**	**Household Covariates**	**Community FE**	**Household + Comm. FE**	**Unadjusted**	**Household Covariates**	**Community FE**	**Household + Comm. FE**
**Height (percentile)**									**Height (HAZ)**								
																	
Wealth disparity	18.2***	5.2**	7.8**	3.6	21.4***	9.8***	14.8***	10.8***	Wealth disparity	0.7***	0.1	0.3**	0.1	0.8***	0.3***	0.6***	0.4***
	[13.0,23.4]	[-1.3,11.7]	[-0.5,16.1]	[-4.9,12.0]	[16.3,26.6]	[3.3,16.4]	[6.6,23.0]	[2.3,19.2]		[0.5,0.9]	[-0.2,0.4]	[-0.1,0.7]	[-0.3,0.5]	[0.6,1.0]	[0.1,0.6]	[0.3,0.9]	[0.1,0.7]
**Height (percentile)**									**Height (HAZ)**								
																	
Schooling disparity	17.2***	4.3*	8.9***	5.9**	19.5***	7.1***	11.7***	8.3***	Schooling disparity	0.7***	0.1	0.4***	0.2*	0.8***	0.3***	0.4***	0.3***
	[12.1,22.2]	[-2.0,10.6]	[2.7,15.1]	[-0.8,12.6]	[14.6,24.5]	[0.9,13.3]	[5.5,17.8]	[1.8,14.8]		[0.4,0.9]	[-0.2,0.4]	[0.1,0.6]	[-0.1,0.5]	[0.6,1.0]	[0.0,0.5]	[0.2,0.7]	[0.1,0.6]
																
**Vocabulary (percentile)**																	
																	
Wealth disparity	27.7***	13.2***	9.5***	2.7	13.4***	1.7	13.6***	7.0**									
	[22.8,32.6]	[6.7,19.7]	[2.3,16.6]	[-4.7,10.2]	[8.2,18.7]	[-5.4,8.8]	[5.1,22.0]	[-1.9,15.9]									
																
**Vocabulary (percentile)**																	
																	
Schooling disparity	26.6***	10.5***	16.4***	12.7***	18.1***	12.1***	14.7***	12.1***									
	[21.8,31.5]	[4.3,16.7]	[11.2,21.5]	[7.0,18.5]	[13.0,23.1]	[5.3,18.9]	[8.4,20.9]	[5.1,19.1]									

*First measurement is age 1 year for height and age 5 years for vocabulary

Each estimate is the coefficient indicating the size of the disparity in a separate regression including the covariates &/or fixed effects

Statistically distinct from 0 at * 0.1 **0.5 ***0.01

Confidence intervals are wide for Community FE in Ethiopia for the wealth disparity because 14 out of 20 communities do not contain households in both top and bottom quartile; communities are more mixed with respect to parental schooling.

## Appendix A

See Appendix Fig. A1, Fig. A2, Fig. A3, Fig. A4, Fig. A5.

See Appendix Table A1, Table A2, Table A3, Table A4, Table A5, Table A6, Table A7, Table A8.

## Footnotes

1 Ethnicities are Amhara, Gurage, Hadiva, Oromo, Sidama, Tigrian, Wolavta, and other (Ethiopia); Scheduled Caste, Scheduled Tribe, Backward Class, and other (India); White, Mestizo including Andean Indigenous, and other (Peru); and H'mong, Kinh and other (Vietnam)
